# Causal association between mitochondrial genes and colorectal cancer: a multi-omics Mendelian randomization study

**DOI:** 10.1007/s12672-025-03699-2

**Published:** 2025-10-14

**Authors:** Zhandong Zhang, Shuaibing Lu, Liangqun Peng, Fusheng Ge, Bin Zhang, Yonglei Zhang, Fei Ma, Yawei Hua, Xiaobing Chen, Wei Yang

**Affiliations:** 1 Department of General Surgery, The Affiliated Cancer Hospital of Zhengzhou University & Henan Cancer Hospital, Zhengzhou, 450008 China; 2 Department of Colorectal Surgery, National Cancer Center/National Clinical Research Center for Cancer/Cancer Hospital, Chinese Academy of Medical Sciences and Peking Union Medical College, Beijing, 100021 China; 3 Department of General Surgery, Louhe Central Hospital, Luohe, 462000 China; 4 Department of Medical Oncology, The Affiliated Cancer Hospital of Zhengzhou University & Henan Cancer Hospital, Zhengzhou, 450008 China; 5 Zhengzhou Key Laboratory for Precision Therapy of Gastrointestinal Cancer, Zhengzhou, 450008 China

**Keywords:** Mitochondrial genes, Colorectal cancer, Multi-omics approaches, Mendelian randomization (MR), Colocalization analysis, Statistical comparison

## Abstract

**Objective:**

Colorectal cancer (CRC) is the leading cause of cancer-related morbidity and mortality globally. Despite the established link between mitochondrial dysfunction and various cancers, including CRC, the precise role of mitochondrial genes remains unclear. This study aimed to elucidate the influence of mitochondrial-related genes on CRC through a multi-omics approach.

**Methods:**

The MitoCarta3.0 database, methylation quantitative trait loci (mQTL), expression QTL (eQTL), and protein QTL (pQTL) data from multiple sources were utilized. CRC-related genetic data were obtained from the IEU OpenGWAS project and FinnGen database. The MR analysis employed five regression models. Integration of the results from three levels of gene regulation revealed significant associations between mitochondrial-related gene regulation and CRC.

**Results:**

We identified 21 genes that exhibit multi-omics evidence associated with CRC. Tier 1 gene PNKD showed significant associations with CRC across multiple omics levels. Tier 2 genes, RBFA, COX15, TXN2, and ACSF3, were linked to CRC at the mQTL-eQTL level. Sixteen tier 3 genes were also identified. A total of eight genes, including COX15, had been identified as potential therapeutic and drug targets. A total of eight genes, including COX15, had been identified as potential drug targets. Additionally, the final structures of the corresponding eight proteins and their respective drugs had been successfully determined.

**Conclusions:**

The multi-omics approach identified several mitochondrial-related genes significantly associated with CRC risk, providing new insights into the role of mitochondrial dysfunction in CRC pathogenesis, and potentially providing further investigation and future therapeutic strategies targeting mitochondrial pathways in CRC management.

**Supplementary Information:**

The online version contains supplementary material available at 10.1007/s12672-025-03699-2.

## Introduction

Colorectal cancer (CRC) is responsible for a significant number of deaths globally, with nearly 900,000 annual fatalities. This represents approximately 10% of the total cases and deaths caused by cancer worldwide [1]. The incidence of CRC among young adults in their 20s and 30s has been progressively increasing recently [2]; however, its underlying etiology remains elusive [3]. Early diagnosis of CRC plays a crucial role in its management. Current treatment options include endoscopic and surgical local excision, radiotherapy, systemic therapy, ablative therapy, and palliative chemotherapy; however, their efficacy in advanced metastatic CRC remains limited [1]. Research on the pathobiology and underlying mechanisms of CRC remains significantly limited, necessitating further investigation to overcome diagnostic and therapeutic challenges [1, 4].

Mitochondria are vital for energy metabolism in cancer cells, and have been implicated in various cancer-related biological processes. Moreover, mitochondrial dysfunction is frequently observed in cancer cells, potentially leading to metabolic reprogramming, which promotes tumor growth and survival [5–8]. Studies have shown that mitochondrial dysfunction is associated with various cancers, including breast, prostate, and lung cancers [9]. For instance, alterations in mitochondrial DNA (mtDNA) and mitochondrial biogenesis have been linked to tumor progression and metastasis in breast cancer [10]. Similarly, mitochondrial gene mutations and changes in mitochondrial dynamics have been implicated in the development and progression of prostate cancer [11].Numerous studies have associated CRC and mitochondrial dysfunction [12, 13]. Basic experiments have demonstrated that CRC cell lines exhibit higher levels of mitochondrial DNA (mtDNA) copy number and function compared to primary colon cells [13], with frequent mtDNA mutations in CRC patients [14–16].Currently, research on the relationship between mitochondria and CRC primarily consists of observational studies, with certain limitations regarding causal inference [17]. Exploration of the causal link between mitochondria and CRC necessitates the implementation of more efficacious research methodologies.

Mendelian randomization (MR) is a robust method for causal inference that uses genetic variants as instrumental variables to assess causal relationships between exposures and outcomes and to mitigate reverse causality, minimize bias, and augment the causal inference capacity of studies. These genetic variants remain constant from conception onwards and remain unaffected by subsequent outcomes or diseases, as well as confounding factors such as sociodemographic characteristics, behavioral patterns, and health conditions [18]. The proliferation of genome-wide association studies (GWAS) and molecular quantitative trait locus (QTL) data provides a robust research foundation for MR research. GWAS leverages genetic associations grounded on single nucleotide polymorphisms (SNPs) and traits to integrate GWAS data with gene expression and methylation data, which offer the potential for identifying expression or methylation QTLs (eQTL or mQTL) [19]. For example, Diabetes and Pancreatic Cancer: Exploring Causal Pathways through Mendelian Randomization Analysis demonstrates the practical utility of MR in behavioral sciences [20]. The Association Between Eczema and Pan-Cancer Risk: A Two-Sample Mendelian Randomization Study highlights the relevance of bidirectional Mendelian randomization in oncology [21].

This study used a two-sample MR approach to examine the underlying causal association between mitochondria-associated genes and CRC using a multi-omics approach. We utilized data from several large-scale databases, including MitoCarta3.0, mQTLs, eQTLs, protein QTLs (pQTLs), and CRC GWAS datasets from the IEU OpenGWAS project and FinnGen. Significant associations between specific mitochondrial genes and CRC risk were identified by integrating the methylation, gene expression, and protein abundance data. Our results revealed that certain mitochondrial genes exhibited consistent and significant causal relationships with CRC across multiple omics layers, providing valuable insights into the particular function of mitochondrial genes in CRC pathogenesis and suggesting a promising avenue for future investigations and identification of potential therapeutic targets.

The remainder of this paper is organized as follows. First, the methods are presented. Thereafter, the results are presented. Finally, the discussions and paper conclusions are presented.

## Methods

### Data source

The mitochondrial-related genes used in this study were acquired from MitoCarta3.0 [22], an extensive repository that provides an updated list of 1136 human mitochondrial genes. Each gene included in MitoCarta3.0 underwent an independent review of the literature to ensure precision and dependability. SNP-CpG associations in blood were identified using DNA mQTLs data from 1980 individuals of European descent, as reported by McRae et al. [21]. Blood eQTLs data were sourced from the eQTLGen Consortium [23], which comprised 31,684 participants. Protein pQTLs were extracted from the UK Biobanks PharmacoProteomics Project (UKB-PPP) [24], involving 54,219 participants from the UKB cohort. Furthermore, GWAS data from a study (Decode) by Ferkingstad et al. [25] on plasma protein levels measured using 4907 aptamers in 35,559 Icelanders provided additional pQTL information. Genetic data associated with CRC were obtained from the IEU OpenGWAS project(https://gwas.mrcieu.ac.uk/)database (19,948 cases and 12,124 controls; dataset number: ebi-a-GCST012879) and FinnGen (https://www.finngen.fi/fi) [26] database (6847 cases and 314,193 controls; dataset number: C3_COLORECTAL_EXALLC).

### Tool variable configuration

**Fig. 1:**
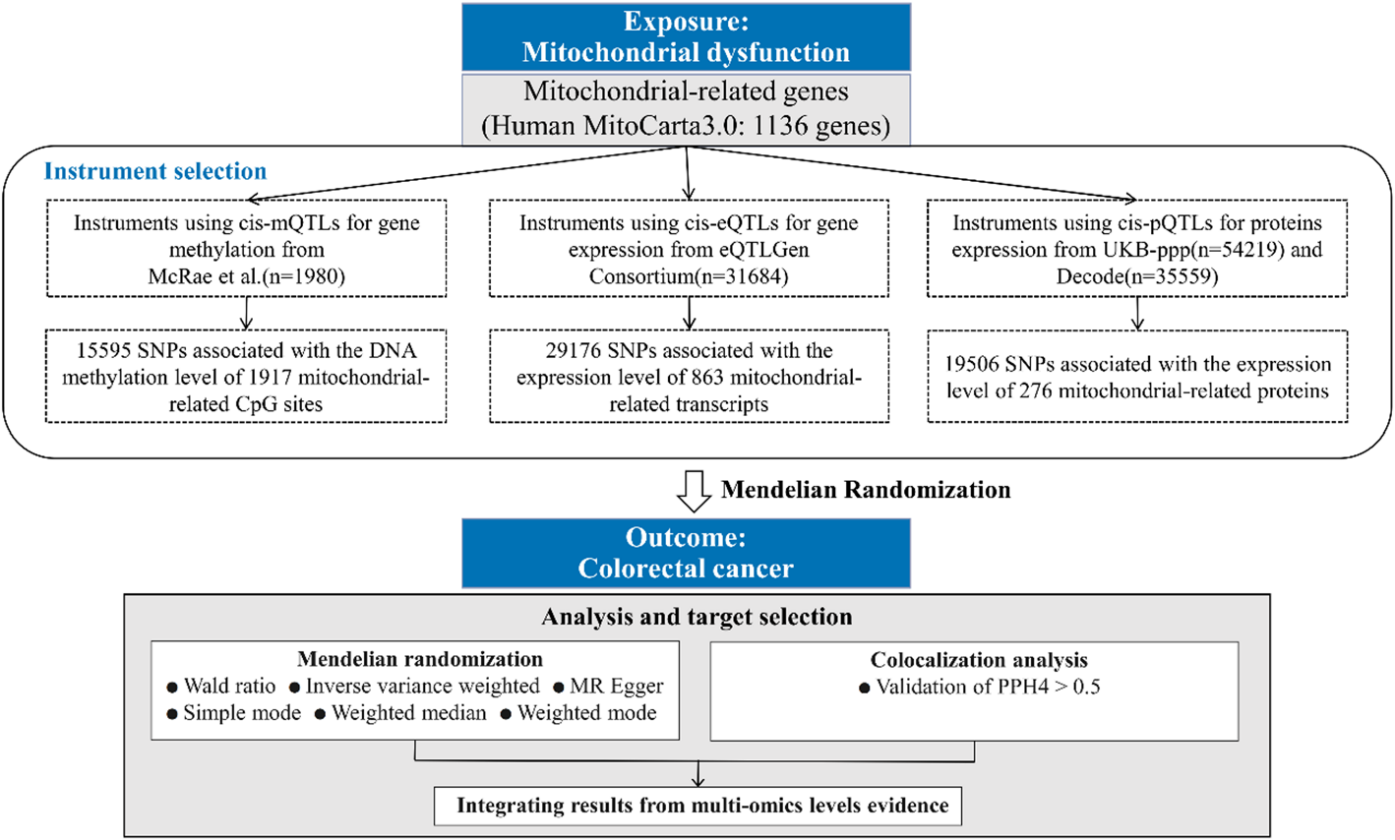
Flow chart of an MR analysis framework for multi-omics evaluation of mitochondrial influence on CRC

Instrumental variables: SNPs located near mitochondrial genes that exhibit significant effects on biomarkers (mQTLs, eQTLs, and pQTLs). The four fundamental assumptions for MR of drug targets:

The following criteria were utilized in this study to screen the instrumental variables:

① Instrumental variables should demonstrate a strong correlation with exposure, indicated by *P* < 5 × 10 ^− 8^ (correlation hypothesis). Additionally, an F-statistic of > 10 was used as the threshold to eliminate weak instrumental variables. ② Instrumental variables should not have a direct impact on the outcome but only influence it through exposure, thereby excluding gene pleiotropy (exclusivity hypothesis). There was no statistically significant difference between the MR-Egger regression intercept term and 0 (*P* > 0.05), and the result of the MR-PRESSO level pleiotropy test was also not significant (*P* > 0.05), indicating the absence of gene pleiotropy. ③ Instrumental variables must be independent of confounders (independent assumptions). Because SNPs selected through MR adhere to genetic principles where parental alleles are randomly assigned to offspring, their susceptibility to environmental factors, such as social, economic, and cultural influences, is minimal or negligible, thus theoretically ensuring independence from these factors. ④ Target correlation: Instrumental variables were restricted to within ± 1000 kb of the cis-acting region of the target gene (Fig. 1).

### Refining instrumental variable filtering

To detect instrumental variables, mQTLs, eQTLs, and pQTLs of mitochondrial genes were screened with a *P*-value threshold of < 5 × 10^−8^, screening hypothesis ①: The linkage disequilibrium coefficient, r^2^, was set to 0.3, the linkage disequilibrium region width to 500 kb, and the secondary allele frequency MAF to > 0.01 to ensure SNP independence and eliminate linkage disequilibrium effects on results. SNPs associated with confounding factors and consequences were excluded using LDtrait (https://ldlink.nih.gov/?tab = ldtrait) [27] (hypotheses ② and ③), whereas those within ± 1000 kb from the cy-acting region of mitochondrial genes were extracted (hypothesis ④). The aforementioned instrumental variables were derived from the mQTLs, eQTLs, and pQTLs data of mitochondrial genes. Relevant SNPs were selected from the GWAS summary data for CRC outcome variables after excluding SNPs with a palindromic structure or direct association with CRC (*P* < 5 × 10^− 8^). Finally, MR-PRESSO was used to eliminate the abnormal SNPs.

### Evaluation of the causal relationship between mitochondria and CRC based on DNA methylation levels (mQTLs), RNA levels (eQTLs), and protein levels (pQTLs)

1. MR analysis based on mQTLs, eQTLs, pQTLs, and CRC.

The study utilized five regression models: MR-Egger regression, inverse variance weighted (IVW) method, weight median method, weighted mode method, and simple mode method. A two-sample MR analysis was conducted exploiting mQTLs, eQTLs, and pQTLs as instrumental variables to evaluate the potential causal relationship between mitochondrial factors and CRC risk. The IVW method is widely employed as the primary approach for estimating causality. When SNPs ≤ 3, the Wald ratio method is used to evaluate the influence of a single SNP on the results; for more than 3 SNPs included, random-effect IVW is applied. The IVW method utilizes multiple SNPs as instrumental variables for estimating causal effects, with the weight of each site determined by the inverse of its variance (R^2). By weighting and summing the causal effect estimates of each site, the final estimate represents the overall causal effect according to IVW. Essentially, MR-Egger uses a weaker hypothesis premise (InSIDE) based on IVW to estimate causal effects. Any deviation caused by pleiotropy in instrumental variables is detected and corrected using regression intercepts to appraise the causal correlation between exposure and outcomes. In cases where horizontal pleiotropy exists, reference can be made to the results obtained from MR-Egger analysis. The random-effect IVW method is used to analyze the causal relationships, whereas the MR-Egger regression approach serves as a complementary analytical tool. Additionally, the false discovery rate (FDR) is a statistical measure used for P-value correction. FDR can be flexibly adjusted to serve as an indicator of test error rate. The FDR can be calculated as $FDR=\frac{{Pvalue*Ran{k_{\hbox{max} }}}}{{{P_{rank}}}}$. The heterogeneity of SNPs was determined using Cochran’s Q test. If the P-value was less than 0.05, the results were considered heterogeneous. I-squared (I²) is another statistical measure of heterogeneity that represents the ratio of total variation attributed to heterogeneity. An I² value greater than 50% indicates significant heterogeneity in IVW results. The formula used to calculate I² is ${I^2}=\frac{{Q - Q\_df}}{Q} \times 100\%$. The intercept term of MR-Egger method and MR-PRESSO were used for pleiotropic analysis, whereas a Leave-one-out approach was ‌exploited for sensitivity analysis. No statistical significance was detected between the intercept term of MR-Egger regression and zero (*P* > 0.05), indicating no evidence of pleiotropy in terms of an insignificant *P*-value (> 0.05) obtained from MR-PRESSO. The SNPs were subjected to Leave-one-out analysis, wherein each SNP was individually excluded and the analysis was re-conducted to assess the effect of each SNP on the overall results. These procedures were executed via the TwoSample MR Package in R 4.1.0, with a predefined significance threshold of α = 0.05.

(2) Utilizing the Steiger filter analysis to examine the causal direction.

The Steiger filter test serves as a statistical tool to confirm the directionality of the causal relationship between the genotype and both the intermediate variables and final outcomes. By leveraging the random allocation of genetic variation, this method estimates the effects of instrumental variables on both intermediate variables and final outcomes. It then calculates the correlation between these two factors to determine whether the causal direction aligns with the expectations. Specifically, this approach computes the interpretive variance of instrumental variable SNPs for mitochondrial CpG sites as well as the variance of outcome variables (CRC). Subsequently, we tested whether the variance of outcomes was smaller than that of the mitochondrial CpG sites. In MR Steiger results, if the variance of outcomes is less than that of mitochondrial CpG sites, this case is judged to be TRUE, indicating a consistent causal relationship in line with the expected direction; conversely, if labeled “FALSE,” it suggests an opposite causal relationship. Next, we applied the same method to examine the causal directions of the eQTLs and pQTLs.

(3) Colocalization analysis.

Colocalization analysis is a technique frequently employed to ascertain whether two phenotypes share the same genetic variation within a specific genomic region, thereby reinforcing the evidence of an association between them. Within a given region, colocalization analysis operates under the assumption that each of the two traits has at most one genuine causal variation, resulting in five distinct and mutually exclusive model assumptions (H0-H4) that encompass all possible associations based on this premise.

H0: There is no significant correlation between phenotype 1 (GWAS) and phenotype 2 (QTL or GWAS) with any SNPs in the genomic region. H1/H2: Both phenotypes 1 and 2 show a significant association with SNPs in a genomic region. H3: Phenotypes 1 and 2 exhibit significant associations with SNP sites in a genomic region; however, these associations are driven by different causal variants. H4: Phenotypes 1 and 2 have significant associations with SNPs in a genomic region attributed to the same causal variant.

In colocalization analysis, a posterior probability (PP.H0-PP.H4) was calculated for each of these models, with the total of these probabilities equal to one. A higher posteriori probability for a specific model suggests that the corresponding model hypothesis is more likely to be valid, given the available data. Generally, we prefer H4 to be valid because it assumes that both traits are influenced by the same causal variation. An H4 probability greater than 0.5 supports this assumption. SNPs within ± 1000 kb from the mitochondrial cis-acting region were extracted using R-package “coloc” for colocalization analysis.

The colocation analysis was conducted based on the mQTLs, eQTLs, and pQTLs.

### Integrating evidence at multiple omics levels

To comprehensively understand the association between mitochondria-related gene regulation and CRC at various levels, we integrated findings from three distinct tiers of gene regulation.

We utilized the following criteria to classify candidate genes into three tiers: ① Tier 1 genes were identified as those demonstrating a causal link with CRC (FDR < 0.05) and having a PP.H4 > 0.5 in at least two of the following levels: methylation, gene expression, and protein abundance; moreover, both gene expression and protein abundance levels were consistent with the direction of the causal association with CRC. ② Tier 2 genes were defined as those displaying a causal association with CRC (FDR < 0.05), excluding tier 1 genes, in terms of any two out of methylation, gene expression, and protein abundance; furthermore, the direction of the causal association was consistent with that observed in CRC cases. Tier 3 genes were designated to have a notable causal relationship (*P* < 0.05) with CRC at any level of methylation, gene expression, and protein abundance.

### Functional enrichment analysis

To investigate the functional characteristics and biological correlations of the genes based on integrating evidence at multiple omics levels, we conducted a comprehensive analysis utilizing functional enrichment methods. Gene Ontology (GO) is a widely recognized framework utilized for the systematic annotation of gene functions, with particular emphasis on molecular functions (MF), biological processes (BP), and cellular components (CC). The top 10 significantly enriched pathways [28] in the BP, CC, and MF enrichment analyses were evaluated based on their visualization P-values. The R package “clusterProfiler” was utilized for GO enrichment analysis, with a significance threshold of *P* < 0.05.

### Constructing a protein–protein interaction (PPI) network

By systematically evaluating and analyzing protein-protein interaction (PPI) networks, researchers can gain deeper insights into the mechanisms by which proteins interact within cellular environments. In this study, the STRING database was utilized to construct a PPI network comprising the genes based on integrating evidence at multiple omics levels, with a minimum required confidence score of 0.4 for interactions [29, 30]. The PPI results were subsequently visualized using Cytoscape version 3.10.3 [31]. Additionally, PPI analysis was conducted using GeneMANIA (https://genemania.org/) [32].

### Identification and prioritization of potential drug candidates molecular Docking simulation

Evaluating protein-drug interactions is crucial for determining the viability of target genes as viable drug targets. The Drug Characteristics Database [33] (DSigDB, Version 1.0, available at http://dsigdb.tanlab.org/DSigD, PMID: 25990557) is an extensive resource comprising 22,527 gene sets and 17,389 distinct compounds, which collectively involve 19,531 genes. This database effectively links drugs and other chemicals to their respective target genes. In this study, the identified target genes based on integrating evidence at multiple omics levels, were uploaded to DSigDB for predicting potential drug candidates to assess the pharmacological activity of these genes. A P-value less than 0.05 was considered statistically significant, with the P-value calculated using Fisher’s exact test, a method that evaluates the significance of the association between categorical variables. The assumption was made that the probability of any gene belonging to any set follows a binomial distribution and is independent. The adjusted P-value was calculated using the Benjamini-Hochberg procedure to control for multiple hypothesis testing.

### Molecular docking simulation

To gain a deeper understanding of the effects of drug candidates on target genes and to assess the pharmacability of these targets, this study conducted atomic-level molecular docking simulations to evaluate the binding energy and interaction modes between drug candidates and their respective targets. Molecular docking simulations provide a robust framework for analyzing the binding affinity and interaction patterns between ligands and their respective drug targets. This study employed the computerized protein-ligand docking software AutoDock Vina 1.1.2 (http://autodock.scripps.edu/) to conduct molecular docking between potential drug candidates and their corresponding target proteins encoded by respective genes. The structural data for the drugs were retrieved from the PubChem Compound Database (https://pubchem.ncbi.nlm.nih.gov/), while the protein structure data were obtained from UniProt (https://www.uniprot.org/). Initially, all water molecules were removed from both the protein and ligand files, followed by the addition of polar hydrogen atoms. The central region of the Grid Box encompassed all domains of the protein, facilitating unrestricted molecular migration. Visualization of molecular docking results was performed using the Discovery Studio 2024 Client.

## Results

### Data source

The data sources consisted exclusively of individuals from Europe, and their details are presented in Table 1. Informed consent was obtained from participants in the original studies, eliminating the need for additional ethics committee approval for this research component. Please refer to Table 1 for further details.

**Table 1:** Brief information of QTLs and GWAS databases in the MR study

Type	Data source	Sample size	Cases	Population
mQTLs	McRae et al.	1980	–	European
eQTLs	eQTLGen	31,684	–	European
pQTLs	UKB-ppp	54,219	–	European
pQTLs	Decode	35,559	–	European
Colorectal cancer	IEU OpenGWAS project	32,072	19,948	European
	FinnGen	321,040	6847	European

### Enhancement of instrumental variables filtering

After applying the aforementioned screening criteria, 23,297 SNPs associated with 1833 CpG sites exhibiting DNA methylation in the mitochondria were identified from cis-mQTL analysis (Supplementary Table 1). Additionally, cis-eQTL analysis revealed that 51,523 SNPs correlated with the expression of 861 transcripts associated with the mitochondria (Supplementary Table 2). Furthermore, by conducting cis-pQTL analysis on two proteomic datasets, we identified 7402 SNPs associated with the expression levels of 138 mitochondria-related proteins (Supplementary Table 3).

### Evaluation of the causal association between mitochondria and CRC based on DNA methylation levels (mQTLs)

1. MR analysis based on cis-mQTLs and CRC.

In this study, we used cis-mQTLs as a genetic tool for MR analysis to systematically investigate the causal link between mitochondria and CRC. The forest plot results are presented in Supplementary Fig. 1. Our MR resonance imaging revealed that 327 CpG sites in the ebi-a-GCST012879 dataset and 303 CpG sites in the finngen_R10_C3_COLORECTAL_EXALLC dataset were significantly associated with CRC. These findings are summarized in Supplementary Table 4. Taking the ebi-a-GCST012879 dataset as an example, the IVW results for CpG site cg00101154 of the MRPL28 gene demonstrated a significant association between cg00101154 and CRC (OR = 1.029, 95%CI: 1.009–1.049, *P* = 0.004, FDR = 0.044). Furthermore, the IVW analysis revealed no heterogeneity in the cis-mQTLs associated with CRC at cg00101154 (I² = 7%, Cochran’s Q = 22.596, *P* = 0.366). The MR-Egger results revealed no statistically significant deviation from zero for the intercept term (*P* = 0.087), and MR-PRESSO detected no substantial horizontal pleiotropy (*P* = 0.342), indicating the robustness of the MR results owing to the absence of horizontal pleiotropy in the SNPs.

(2) Utilizing Steiger filter analysis to examine the causal direction.

The results of the Steiger orientation test in Supplementary Table 4 indicate that the direction of mitochondrial CpG sites in the CRC dataset was “TRUE,” signifying a consistent causal relationship between mitochondrial CpG sites and the outcome.

(3) Colocalization analysis.

The colocalization analysis, based on the CRC dataset and mitochondrial mQTLs, revealed that a PP.H4 value greater than 0.8 suggested strong evidence of colocalization between the two traits in this region. A PP.H4 value between 0.5 and 0.8 indicated moderate evidence of colocalization for the two traits in this region (Supplementary Table 4). Taking the ebi-a-GCST012879 dataset as an example (Supplementary Table 4), MRPS26(cg24504518) demonstrated a PP.H4 value higher than 0.8, indicating a shared causal variation in this region. Similar explanations can be found in other results.

### Assessment of the causal link between mitochondria and CRC based on RNA levels (eQTLs)

1. MR analysis based on cis-eQTLs and CRC.

In this study, we used cis-eQTLs as a genetic tool for MR analysis to systematically evaluate the causal relationship between mitochondria and CRC. The forest plot results are presented in Supplementary Fig. 2. After conducting an MR analysis of mitochondria and CRC, 218 mitochondrial genes were significantly linked to CRC in the ebi-a-GCST012879 dataset, and 263 mitochondrial genes were significantly associated with CRC in the finngen_R10_C3_COLORECTAL_EXALLC dataset. The results are summarized in Supplementary Table 5. Using the ebi-a-GCST012879 dataset as an example, IVW analysis revealed that ABHD11 was associated with mitochondria and CRC (OR = 1.096, 95%CI: 1.052–1.142, *P* < 0.001, FDR < 0.001). Furthermore, no heterogeneity was observed in the mitochondrial cis-eQTLs associated with CRC based on the IVW results (I² = 0%, Cochran’s Q = 44.718, *P* = 0.647). MR-Egger analysis indicated no statistically significant deviation from zero for the intercept term (*P* = 0.074), whereas MR-PRESSO did not identify any substantial horizontal pleiotropy (*P* = 0.660), suggesting that SNPs do not exhibit horizontal pleiotropy and confirming the robustness of the MR results.

(2) Utilizing Steiger filter analysis to examine the causal direction.

The results of the Steiger orientation test in Supplementary Table 5 demonstrated that the direction of mitochondrial genes in the CRC dataset was “TRUE,” indicating a consistent causal relationship between mitochondria and outcome.

(3) Colocalization analysis.

The colocalization analysis of eQTLs based on the CRC datasets and mitochondrial genes revealed strong colocalization evidence between the two traits in a region when PP.H4 > 0.8, whereas moderate colocalization evidence was suggested when PP.H4 values ranged between 0.5 and 0.8, as shown in Supplementary Table 5. For instance, in the ebi-a-GCST012879 dataset, LIPT2’s PP.H4 value of 0.695 indicates medium colocalization evidence of shared causal variation in this region.

### Evaluation of the causal association between mitochondria-associated proteins and CRC based on protein levels (pQTLs)

1. MR analysis using cis-pQTLs and CRC.

As a genetic tool for MR analysis, cis-pQTLs were used to systematically assess the causal impact of mitochondria-associated proteins on CRC (Supplementary Fig. 3). After conducting MR analysis of mitochondria-associated proteins and CRC, we discovered 43 proteins linked to CRC in the ebi-a-GCST012879 dataset and 38 proteins associated with CRC in the finngen_R10_C3_COLORECTAL_EXALLC dataset (results are presented in Supplementary Table 6). Using the ebi-a-GCST012879 dataset as an example, IVW analysis revealed a significant correlation between ALDH2 protein and CRC (OR = 0.601, 95%CI: 0.452–0.799, *P* < 0.001, FDR = 0.009). Furthermore, IVW analysis demonstrated no heterogeneity between ALDH2 protein and cis-pQTLs associated with CRC (I² = 0%, Cochran’s Q = 0.293, *P* = 0.961). The MR-Egger regression indicated that there was no statistically significant intercept term compared to zero (*P* = 0.644), whereas MR-PRESSO did not detect any significant horizontal pleiotropy (*P* = 0.973). These findings suggest that there was no evidence of horizontal pleiotropy among the SNPs, reinforcing the robustness of the MR findings.

(2) Utilizing Steiger filter analysis to examine the causal direction.

The results of the Steiger orientation test (presented in Supplementary Table 6) demonstrated that the direction of mitochondria-associated proteins in the CRC dataset was consistently “TRUE,” indicating a reliable causal relationship between mitochondria-associated proteins and the outcome.

(3) Colocalization analysis.

The results of the colocalization analysis based on the pQTLs of mitochondria-related genes and CRC revealed that a PP.H4 value greater than 0.8 indicates strong evidence of colocalization between the two traits in this region. A PP.H4 value between 0.5 and 0.8 suggested moderate evidence of colocalization for the two traits in this region (refer to Supplementary Table 6 for detailed results). Examining the ebi-a-GCST012879 dataset as an example, a PP.H4 value less than 0.5 for FDX1 implied no shared causal variation in this region.

### Integrating evidence at multiple omics levels

**Fig. 2:**
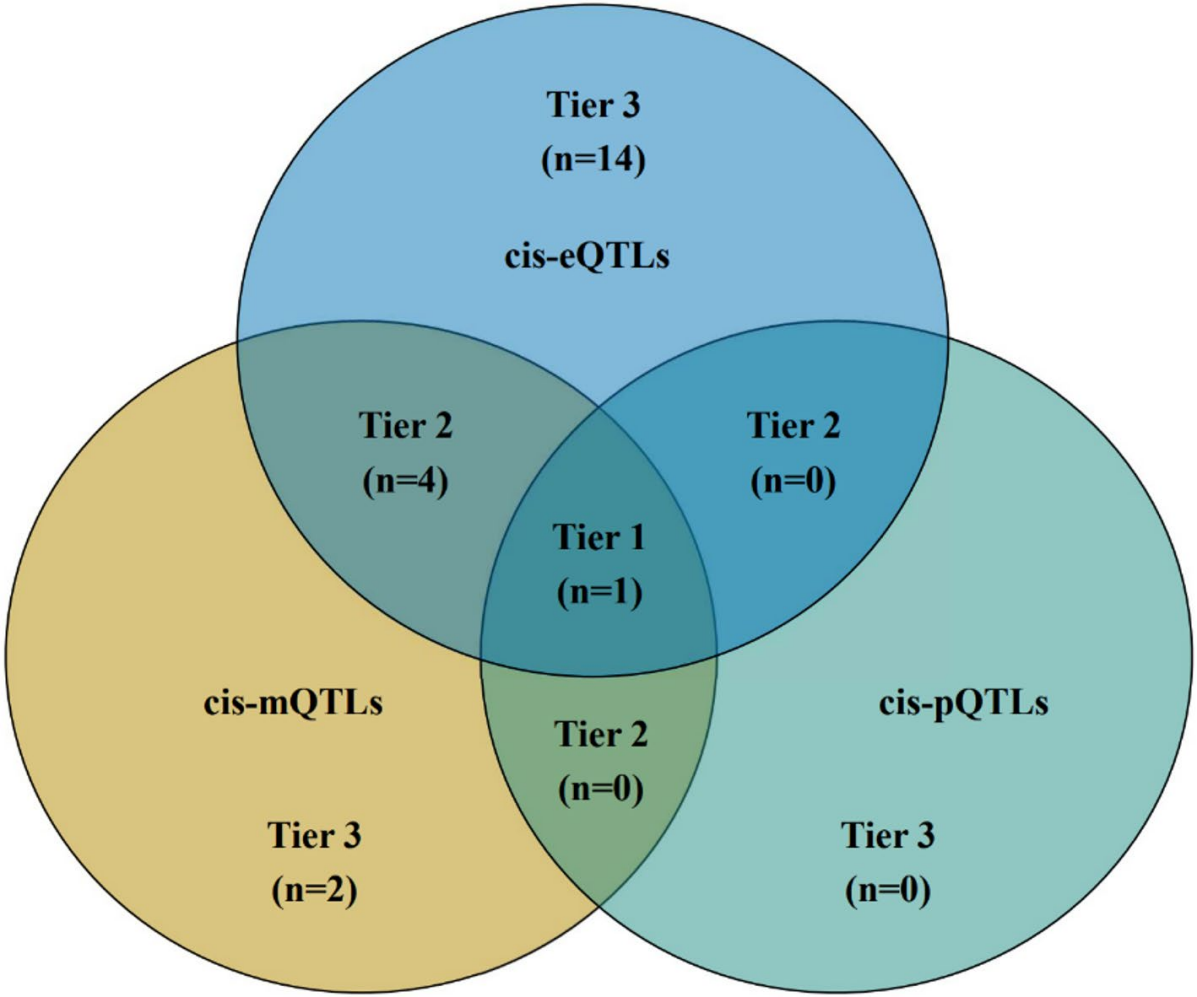
Grading result of CRC candidate genes after integrating the evidence from various omics levels

**Fig. 3:**
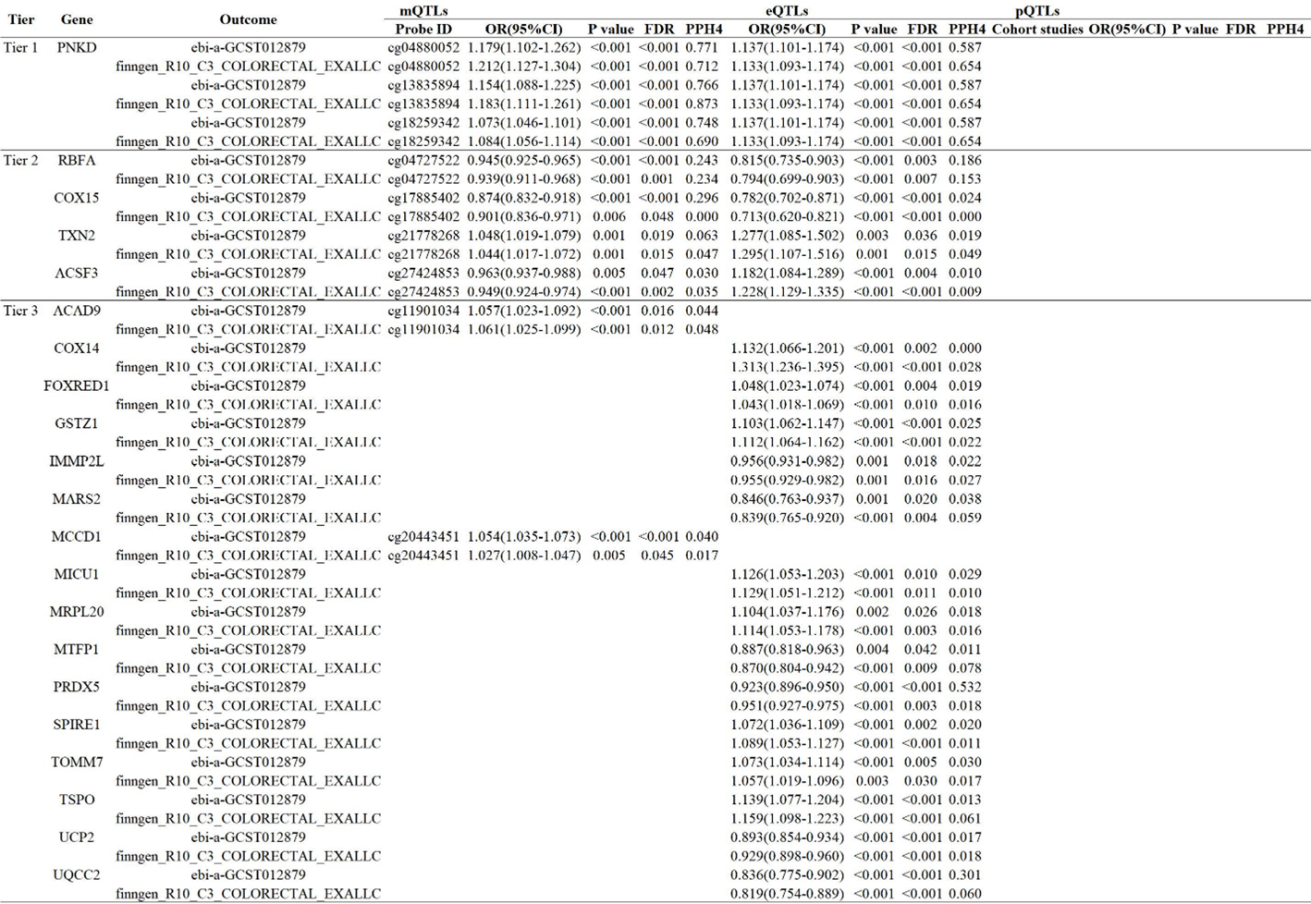
Genetic prediction of methylation, expression, and proteins of candidate genes in CRC

The findings after integrating the evidence from various omics levels are presented in Fig. 2. paroxysmal nonkinesigenic dyskinesia (PNKD) was identified as a tier 1 gene, supported by primary evidence from multiple omics sources ( Fig. 3 for detailed results). At the mQTL-eQTL level, PNKD was causally linked to CRC. Additionally, RBFA, COX15, TXN2, and ACSF3 were identified as tier 2 genes associated with CRC at the mQTL-eQTL level. Furthermore, we identified 16 tier 3 genes associated with CRC: ACAD9, COX14, FOXRED1, GSTZ1, IMMP2L, MARS2, MCCD1, MICU1, MRPL20, MTFP1, PRDX5, SPIRE1, TOMM7, TSPO, UCP2, and UQCC2. Among these tertiary genes, positive associations were observed between cg11901034 methylation of ACAD9 and CRC risk as well as COX14 gene expression levels.

### Functional enrichment analysis

**Fig. 4:**
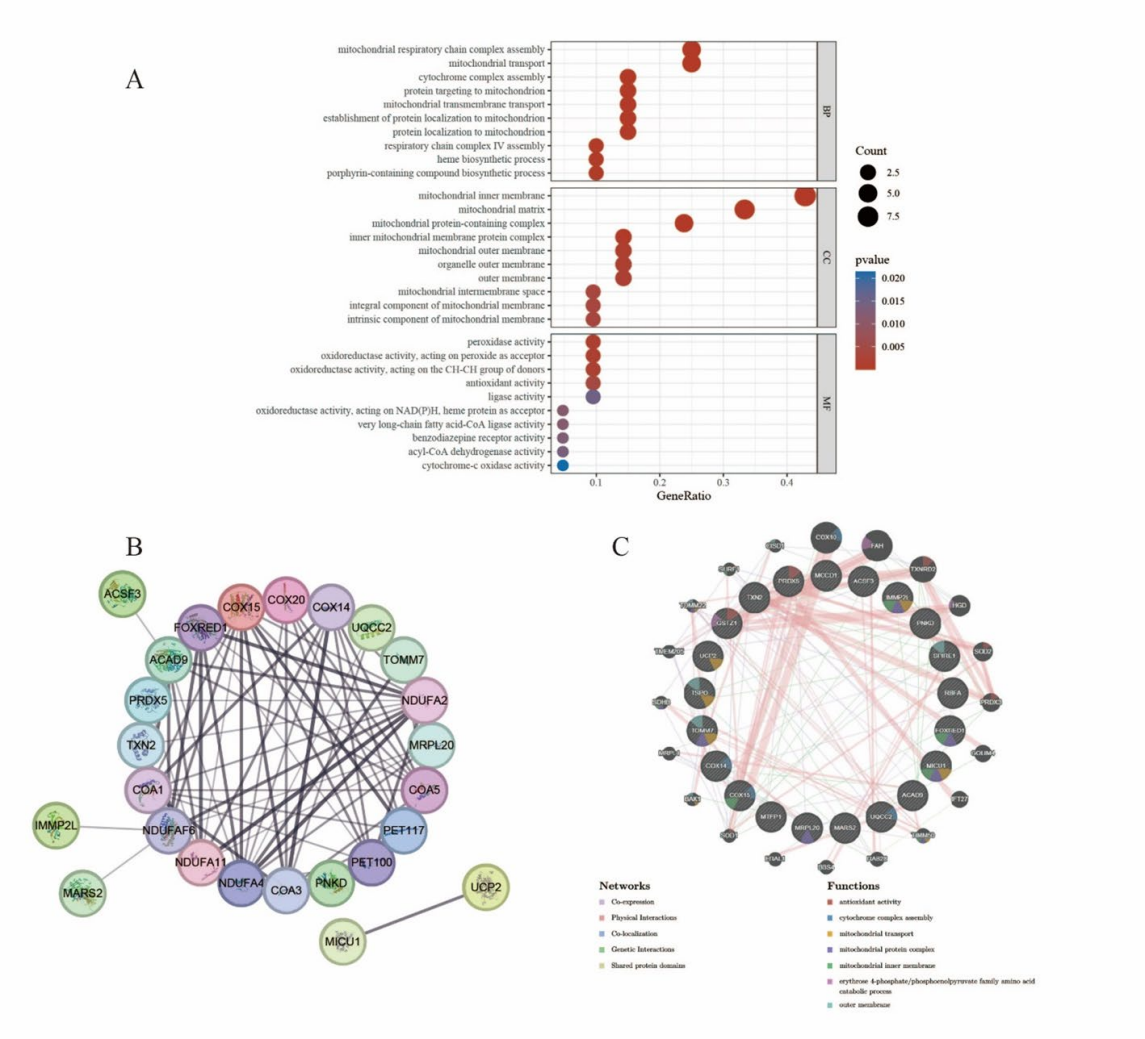
Biological processes and protein-protein Interaction (PPI) network were identified by gene ontology and the STRING database. **A** GO Enrichment Analysis Results. **B** Constructing a PPI network with STRING, detailed display of the network node information, connection line information, and the composition of the different sub-network information. **C** The PPI network was constructed using GeneMANIA. (A variety of colors are employed within the circles to denote the specific functional pathways associated with each gene.)

GO enrichment analysis revealed that BP associated with the 21 genes were predominantly involved in mitochondrial respiratory chain complex assembly, mitochondrial transport and cytochrome complex assembly. MF primarily pertained to the mitochondrial inner membrane, mitochondrial matrix as well as mitochondrial protein-containing complex. CC involved were primarily associated with peroxidase activity, and oxidoreductase activity, acting on peroxide as acceptor. (Fig. 4A, Table S7).

### Protein–protein interaction (PPI) networks of core genes

Through STRING analysis, a PPI network comprising 25 nodes and 85 edges was constructed, illustrating the interactions between 15 target genes and their associated genes (Fig. 4B, Table S8). The degree centrality of each node was assessed using Cytoscape. The analysis revealed that among the 21 genes of interest, COX15 (Degree = 12) and COX14 (Degree = 11) exhibited the highest degree centrality.

Through GeneMANIA analysis, we identified 20 potential interacting genes associated with the 21 target genes, resulting in a PPI network comprising 159 interaction pairs. These interactions were categorized as follows: Physical Interactions (38.9%), Co-expression (32.7%), and Genetic Interactions (19.4%). The functional analysis of this network elucidates the roles of drug targets and associated genes, focusing on their key functions such as antioxidant activity, assembly of cytochrome complexes, mitochondrial transport, mitochondrial protein complexes, mitochondrial inner membrane processes, catabolic processes of erythrose 4-phosphate/phosphoenolpyruvate family amino acids, and outer membrane functions (Fig. 4C, Table S9).

### Assessment of the druggability of core genes

**Fig. 5:**
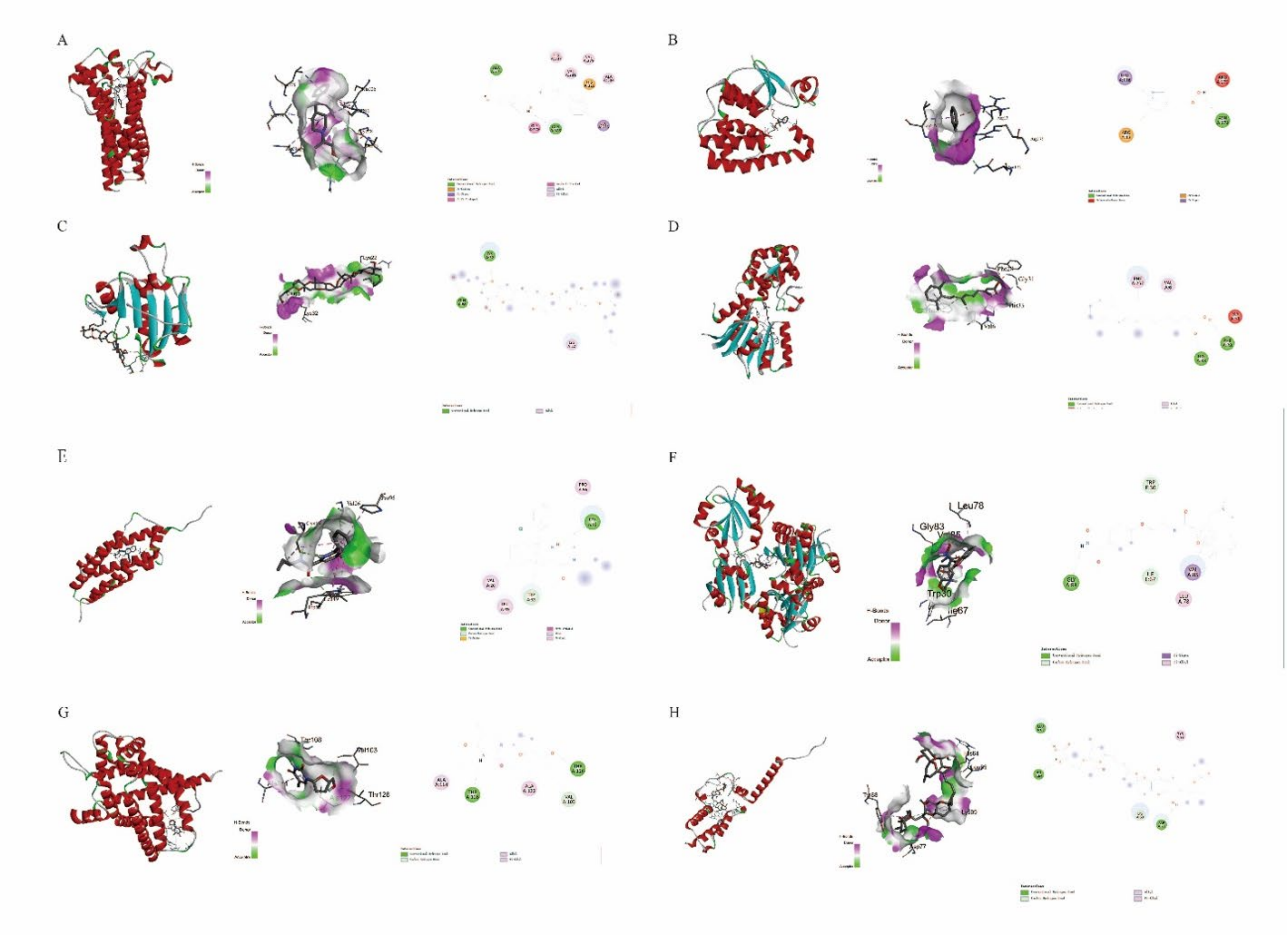
The complete docking results for eight protein-drug pairs were obtained based on the derived interaction binding energies. **A** COX15 (target)_ifenprodil (drug); **B** GSTZ1_CUMENE_HYDROPEROXIDE; **C** PRDX5_Gambierol; **D** RBFA_tretinoin; **E** TSPO_PK_11195; **F** TXN2_PCI-24,781; **G** UCP2_stavudine; **H** UQCC2_okadaic_acid

The DSigDB database was utilized to predict potential effective intervention drugs. Based on an adjusted p-value threshold of less than 0.05, the candidate drug with the smallest adjusted p-value that demonstrated clinical therapeutic potential was identified for each gene [34] (Table 2). In total, eight genes satisfied these criteria. The final outcome included the structural configurations of eight proteins and eight pharmaceutical compounds, with their IDs listed in Table 3. The binding energy for each interaction was calculated, yielding comprehensive docking results for a total of eight protein-drug pairs (Table 3; Fig. 5). Each drug candidate binded to its respective protein target via observable hydrogen bonding and robust electrostatic interactions, exhibiting high affinity (binding energy less than − 5 kcal/mol). The molecular docking results obtained based on this method have a high degree of credibility [35].

**Table 2:** Prediction of drug candidates utilizing DSigDB

Genes	Drug names	*P* value	Adjusted *P* value
COX15	ifenprodil	0.001	0.026
GSTZ1	CUMENE HYDROPEROXIDE	0.001	0.014
PRDX5	Gambierol	0.001	0.009
RBFA	tretinoin	0.003	0.031
TSPO	PK 11,195	0.001	0.016
TXN2	SODIUM CHROMATE	0.001	0.013
UCP2	stavudine	0.001	0.020
UQCC2	okadaic acid	0.005	0.035

**Table 3:** Docking results of available proteins with small molecules

Target	UniProt ID	Identifier	Drug	PubChem ID	Affinity(kcal/moL)
COX15	Q7KZN9	AF-Q7KZN9-F1	ifenprodil	3689	-9.4
GSTZ1	O43708	1FW1	CUMENE HYDROPEROXIDE	6629	-6.3
PRDX5	P30044	3MNG	Gambierol	6,442,244	-7.4
RBFA	Q8N0V3	8IPL	tretinoin	444,795	-8.1
TSPO	P30536	AF-P30536-F1	PK 11,195	1345	-7.8
TXN2	Q99757	1W4V	PCI-24,781	11,749,858	-7.3
UCP2	P55851	AF-P55851-F1	stavudine	18,283	-5.9
UQCC2	Q9BRT2	AF-Q9BRT2-F1	okadaic acid	446,512	-8.5

## Discussion

CRC is a prevalent malignancy with significant global health implications and ranks among the leading causes of cancer-related deaths worldwide. Known for its high morbidity and mortality rates, CRC severely affects the quality of life of patients and imposes substantial healthcare burdens [36]. The need for improved understanding and management of CRC is underscored by its complex etiology, which involves genetic, environmental, and lifestyle factors [37]. Recent advancements in high-throughput technologies have facilitated the integration of genomics, transcriptomics, and proteomics, thereby enhancing our ability to investigate the multifaceted nature of CRC [38]. We investigated the biological causal relationships between mitochondrial genes and colorectal cancer (CRC) using a multi-omics approach. By integrating data from diverse sources, including mQTLs, eQTLs, and pQTLs, we aimed to identify potential biomarkers and therapeutic targets for CRC. The utilization of MR and colocalization analysis bolsters the robustness of the findings, potentially leading to substantial advancements in CRC diagnosis and treatment. This approach provides a promising direction for personalized medicine by enabling more precise and effective interventions based on the genetic and molecular profiles of an individual.

Mitochondrial dysfunction is increasingly being acknowledged as a hallmark of cancer progression, with changes in mitochondrial DNA (mtDNA) and associated genes playing pivotal roles [39]. Additionally, mutations in mitochondrial tRNA genes, such as the A12308G alteration in tRNA(Leu(CUN)), have been proposed as biomarkers for CRC [40]. Mitochondrial genome instability, characterized by increased heteroplasmy and mtDNA copy number variations, has been observed in CRC tissues, implicating these alterations in tumorigenesis [41]. The interplay between the nuclear and mitochondrial genomes also plays a pivotal role in CRC. For instance, mutations in the nuclear-encoded polymerase gamma (POLG) gene, which is essential for mtDNA maintenance, are associated with reduced mtDNA content and mitochondrial dysfunction in CRC [42]. Collectively, these findings emphasize the multifaceted role of mitochondrial genes and their regulatory pathways in CRC. By elucidating these mechanisms, this study provides a comprehensive understanding of the biological underpinnings of CRC and identifies potential targets for therapeutic intervention.

Our study reveals significant associations between mitochondrial genes and CRC, such as ABHD11 and ALDH2. The abhydrolase domain containing 11(ABHD11)gene has been implicated in the progression of CRC through its association with the integrin subunit alpha 5 (ITGA5)/focal adhesion kinase (FAK)/phosphoinositide 3 kinase (PI3K)/Akt signaling pathway. Long non-coding RNA (lncRNA) ABHD11-AS1, transcribed from the ABHD11 gene locus, has been shown to be overexpressed in CRC tissues and is associated with poor prognosis. Functional studies have demonstrated that ABHD11-AS1 knockdown significantly reduces CRC cell proliferation, migration, and invasion while inducing apoptosis [43]. The ABHD11 gene’s association with CRC further supports the involvement of mitochondrial lipid metabolism in cancer, which is consistent with the findings of Zhang et al., who highlighted mitochondrial dysfunction in colitis-associated dysplasia, a precursor of CRC [44]. The role of ABHD11 in essential signaling pathways underscores its potential as a therapeutic target for CRC treatment. Aldehyde dehydrogenase 2 (ALDH2), a pivotal mitochondrial enzyme involved in the oxidative metabolism of alcohol, plays a dual role in CRC progression. Wei et al. demonstrated that ALDH2 enhances cancer stemness and metastasis in CRC by activating β-catenin signaling, corroborating our findings [45]. Furthermore, ALDH2 mediates alcohol-induced immune escape in CRC by stabilizing the expression of programmed cell death ligand 1 (PD-L1), thereby enabling tumors to evade immune surveillance [46]. These observations indicate that ALDH2 is a key regulator of both CRC progression and immune evasion.

Through the integration of multi-omics evidence, PNKD has been recognized as a tier 1 gene. In addition, RBFA, COX15, TXN2, and ACSF3 have been identified as tier 2 genes.

Recent studies have emphasized the role of DNA methylation at CpG sites near the PNKD gene in modulating CRC risk. For instance, methylation at the CpG site cg13835894, located near the PNKD gene, has been associated with an increased risk of CRC, suggesting that epigenetic modifications in this region may contribute to tumorigenesis [47]. A comprehensive analysis of seven independent GWAS identified a previously unknown CRC risk locus in the 2q35 region, specifically rs992157. This genetic region is notably located within the intronic regions of the PNKD and TMBIM1 genes [48]. Another study demonstrated a significant association between the genetic variant rs992157-PNKD1/TMBIM1 and Serrated Polyposis Syndrome, which increases susceptibility to CRC [49]. These findings highlight the importance of epigenetic factors in CRC development and suggest that PNKD may serve as a potential biomarker for early detection and prognosis.

Ribosome-binding factor A (RBFA) is a mitochondrial protein involved in ribosome biogenesis. It plays an essential role in the maturation of small ribosomal subunits by binding to the 16 S rRNA and facilitating proper folding and processing [50]. Recent studies have indicated that the interaction of RBFA with ribosomal proteins and their regulation by ribosome-associated GTPases are vital for maintaining the fidelity of protein synthesis, particularly under stressful conditions [51]. Cytochrome c oxidase 15 (COX15) encodes heme a synthase, an enzyme involved in the biosynthesis of heme a, a critical component of cytochrome c oxidase (complex IV) in the mitochondrial respiratory chain [52]. Mutations in COX15 have been linked to various mitochondrial disorders, including Leigh syndrome and hypertrophic cardiomyopathy [53]. Thioredoxin 2 (TXN2) is a mitochondrial redox protein that is essential for maintaining cellular redox homeostasis and protecting cells against oxidative stress [54]. The function of TXN2 in modulating mitochondrial reactive oxygen species and its protective role in apoptosis underscore its importance in cellular stress responses [55]. ACSF3 encodes acyl-CoA synthetase family member 3, which is crucial for the mitochondrial synthesis of malonyl-CoA from malonate [56]. This pathway is vital for mitochondrial fatty acid biosynthesis and energy metabolism. Mutations in ACSF3 cause combined malonic and methylmalonic aciduria (CMAMMA), a metabolic disorder characterized by elevated levels of malonic and methylmalonic acids [57].

This study has several advantages. First, the utilization of MR and colocalization techniques, which leverage genetic variations to estimate the causal effects of mitochondrial gene methylation, expression, and protein abundance, is noteworthy. The MR approach mitigates the bias from confounding factors and reverse causation, thereby enhancing causal inferences. Colocalization methods further reduce the potential biases arising from linkage disequilibrium. Second, the Steiger directionality test confirmed that all pertinent SNPs showed the expected causal direction, bolstering the robustness of our MR analysis and reinforcing the credibility of our causal inferences. Employing Steiger filtering in genetic epidemiology is crucial for ensuring the reliability of causal direction conclusions. Third, the integration of multi-omics data provides a holistic understanding of the roles of mitochondrial genes in CRC. This methodological approach captured the complexity of gene regulation and its impact on disease risk. The identified genes are potential candidates for further mechanistic studies and clinical applications, leading to the development of multifaceted diagnostic and therapeutic strategies. Finally, the substantial sample size of the GWAS enhanced the statistical power of our analysis. Furthermore, the consistency of our results across multiple datasets supported our findings.

The results of the functional enrichment analysis indicated that the 21 identified genes are predominantly associated with critical biological processes, including the assembly of mitochondrial complexes and mitochondrial transport. These processes are fundamental to cellular metabolism and energy production. The significantly enriched pathways highlight the potential roles of these genes in resisting apoptosis and regulating metabolic reprogramming, offering novel insights into the pathogenesis of colorectal cancer [58]. In the construction of the protein-protein interaction (PPI) network, the degree values of COX15 and COX14 were notably elevated, indicating that these two genes occupy central positions within the network and suggesting that these genes may play pivotal roles in the biological processes associated with colorectal cancer [59].

By assessing the druggability of core targets, we identified eight drug candidates exhibiting favorable affinity towards the core genes. The identification of these compounds offers novel avenues for the development of innovative therapeutic strategies aimed at targeting mitochondrial genes. Future research should focus on validating the clinical effectiveness of these drug candidates and elucidating their mechanisms of interaction with tumor targets to facilitate the advancement of effective treatments for colorectal cancer [60]. These findings provide a robust theoretical foundation for drug design and development, potentially enhancing the efficacy of targeted therapies in clinical applications [61].

To further verify the functional significance of candidate genes identified through multi-omics and MR analysis in CRC, in vivo mouse model experiments may be conducted. For example, a subcutaneous xenograft model can be established in 5-week-old female BALB/c nude mice to compare differences in tumor formation between control and gene intervention groups. Tumor volume should be measured regularly, and molecular analysis performed on endpoint tissue samples. Furthermore, orthotopic transplantation or metastasis models can be used to observe tumor growth and metastasis, while assessing mitochondrial function and related pathways [62].

This study has several limitations that merit consideration. .First, the QTL and GWAS data used were primarily derived from European populations, which may limit the generalizability of the findings to other ethnicities or regions. Future studies should validate these findings in diverse ethnic and regional populations to ensure broader applicability. Second, this study lacks validation in independent cohorts, so the robustness of the findings still needs further confirmation in larger, more diverse populations and datasets. Additionally, the QTL datasets were derived from different research platforms and detection technologies. Although these data underwent rigorous quality control, batch effects or inter-platform differences may still affect causal inferences. Lastly, the molecular docking analysis in this study was based on rigid-body assumptions and did not fully consider protein conformational flexibility and molecular dynamics under physiological conditions, which represents an oversimplification. Future studies could employ more sophisticated computational approaches, such as molecular dynamics simulations, for validation. In summary, future research should perform independent validation in multi-ethnic populations and prospective cohorts, alongside experimental studies to further substantiate causal relationships, thereby enhancing the robustness and clinical translational value of the conclusions.

## Conclusions

In summary, we systematically investigated the intricate biological connections between mitochondrial genes and CRC using MR combined with multi-omics data. We identified various potential novel targets that offer fresh insights into the mechanisms underlying CRC pathogenesis. These findings advance our understanding of CRC and establish a foundation for the development of innovative diagnostic and therapeutic strategies. The integration of extensive genomic data and robust statistical methods bolstered causal inferences between mitochondrial genes and CRC risk, providing a solid basis for future research in this field.

## Supplementary Information

Additional file 1: Supplementary Table 1 Basic information of some SNPs associated with cis-mQTL and colorectal cancer. Supplementary Table 2 Basic information of some SNPs associated with cis-eQTL and colorectal cancer. Supplementary Table 3 Basic information of some SNPs associated with cis-pQTL and colorectal cancer. Supplementary Table 4 MR results of the association between mitochondrial gene methylation and colorectal cancer. Supplementary Table 5 MR results illustrating the association between mitochondrial gene expression and colorectal cancer. Supplementary Table 6 MR results showing the association between levels of mitochondria-associated proteins and colorectal cancer. Supplementary Table 7 Detailed GO results. Supplementary Table 8 Detailed STRING results. Supplementary Table 9 Detailed genemania-network and genemania-functions. Supplementary Fig. 1 Forest plot of the association between methylation of mitochondrial genes and CRC. Supplementary Fig. 2 Forest map illustrating the association between mitochondrial gene expression and CRC. Supplementary Fig. 3 Forest map illustrating the association between levels of mitochondria-associated proteins and CRC.

## Data Availability

The datasets for this study can be found in the MitoCarta3.0; the IEU OpenGWAS project（https://gwas.mrcieu.ac.uk/）database as well as FinnGen（https://www.finngen.fi/fi）. The original contributions presented in the study are included in the article/Supplementary Material, further inquiries can be directed to the corresponding authors.
