# Supplementary figures and images for: Causal association between mitochondrial genes and colorectal cancer: a multi-omics Mendelian randomization study
Source: Discov Oncol. 2025 Oct 14;16:1864. doi: 10.1007/s12672-025-03699-2 (PMC12521712; doi:10.1007/s12672-025-03699-2)

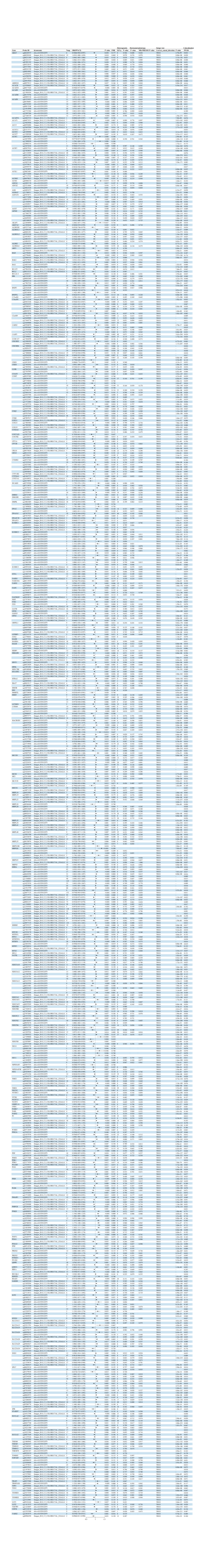

Supplement: Supplementary file 1 — Additional file 1: Supplementary Table 1 Basic information of some SNPs associated with cis-mQTL and colorectal cancer. Supplementary Table 2 Basic information of some SNPs associated with cis-eQTL and colorectal cancer. Supplementary Table 3 Basic information of some SNPs associated with cis-pQTL and colorectal cancer. Supplementary Table 4 MR results of the association between mitochondrial gene methylation and colorectal cancer. Supplementary Table 5 MR results illustrating the association between mitochondrial gene expression and colorectal cancer. Supplementary Table 6 MR results showing the association between levels of mitochondria-associated proteins and colorectal cancer. Supplementary Table 7 Detailed GO results. Supplementary Table 8 Detailed STRING results. Supplementary Table 9 Detailed genemania-network and genemania-functions. Supplementary Fig. 1 Forest plot of the association between methylation of mitochondrial genes and CRC. Supplementary Fig. 2 Forest map illustrating the association between mitochondrial gene expression and CRC. Supplementary Fig. 3 Forest map illustrating the association between levels of mitochondria-associated proteins and CRC. [file 12672_2025_3699_MOESM1_ESM.zip › 【Supplementary files】/Supplementary Figure 1 Forest plot of the association between methylation of mitochondrial genes and CRC.png]

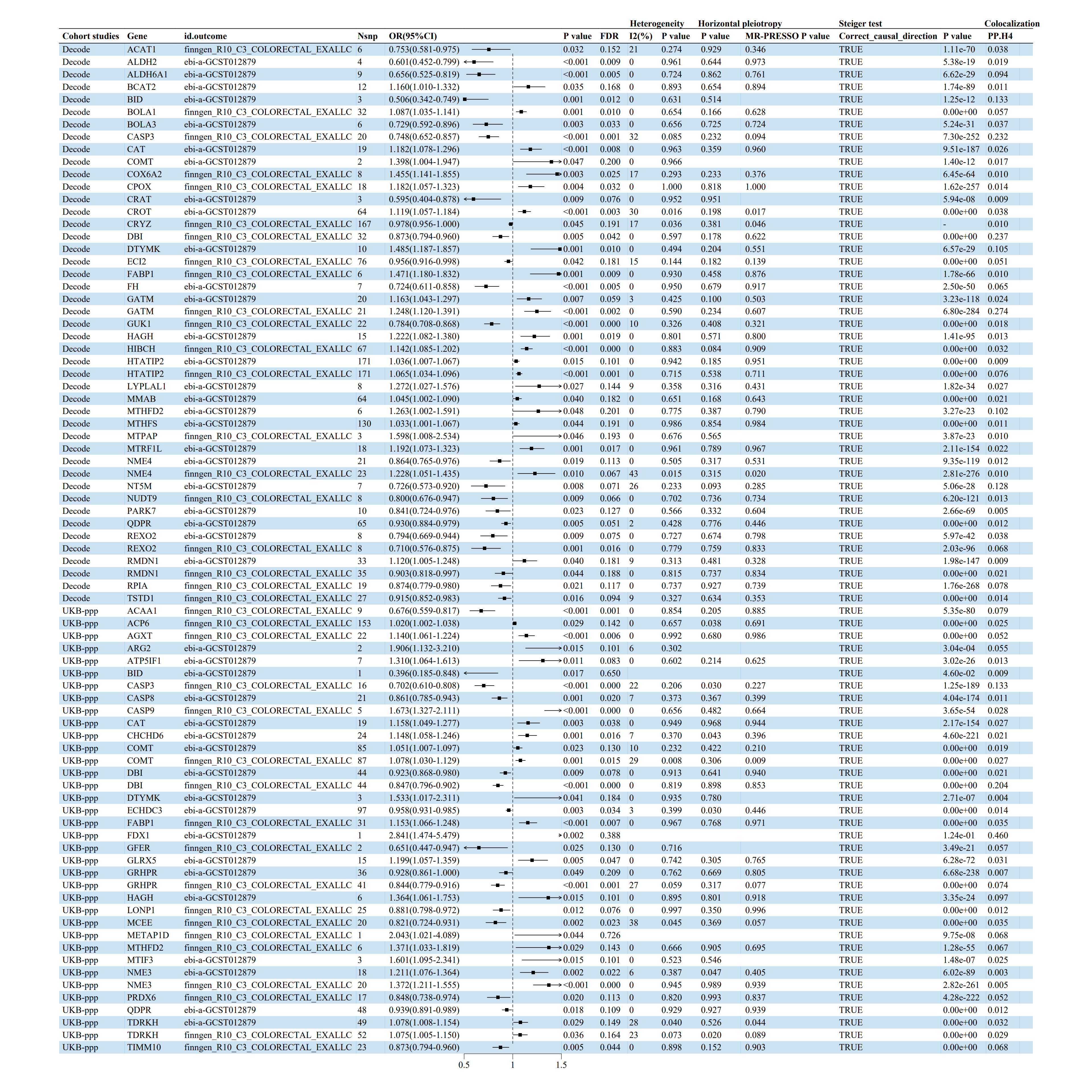

Supplement: Supplementary file 1 — Additional file 1: Supplementary Table 1 Basic information of some SNPs associated with cis-mQTL and colorectal cancer. Supplementary Table 2 Basic information of some SNPs associated with cis-eQTL and colorectal cancer. Supplementary Table 3 Basic information of some SNPs associated with cis-pQTL and colorectal cancer. Supplementary Table 4 MR results of the association between mitochondrial gene methylation and colorectal cancer. Supplementary Table 5 MR results illustrating the association between mitochondrial gene expression and colorectal cancer. Supplementary Table 6 MR results showing the association between levels of mitochondria-associated proteins and colorectal cancer. Supplementary Table 7 Detailed GO results. Supplementary Table 8 Detailed STRING results. Supplementary Table 9 Detailed genemania-network and genemania-functions. Supplementary Fig. 1 Forest plot of the association between methylation of mitochondrial genes and CRC. Supplementary Fig. 2 Forest map illustrating the association between mitochondrial gene expression and CRC. Supplementary Fig. 3 Forest map illustrating the association between levels of mitochondria-associated proteins and CRC. [file 12672_2025_3699_MOESM1_ESM.zip › 【Supplementary files】/Supplementary Figure 3 Forest map illustrating the association between levels of mitochondria-associated proteins and CRC.png]
